# Molecular Analyses of Aspirated Cystic Fluid for the Differential Diagnosis of Cystic Lesions of the Pancreas: A Systematic Review and Meta-Analysis

**DOI:** 10.1155/2016/3546085

**Published:** 2015-12-24

**Authors:** Xiaorong Guo, Xianbao Zhan, Zhaoshen Li

**Affiliations:** Department of Gastroenterology, Changhai Hospital, Second Military Medical University, Shanghai 200433, China

## Abstract

*Background*. Researchers have evaluated various molecular tests for improving the differential diagnosis of cystic lesions of the pancreas. *Methods*. Six electronic databases were searched for articles on molecular tests for the diagnosis of pancreatic cysts. Measures of accuracy were extracted from selected articles and pooled by the random-effects model. Summary receiver operating characteristic curves were used to analyze the overall accuracy of the molecular tests. Pooled sensitivity and specificity values [95% confidence intervals] are reported. *Results*. The systematic review included eight studies of 428 patients in total. We determined the sensitivities and specificities of tests for *KRAS* mutations (0.47 [0.39–0.54], 0.98 [0.93–0.99]) and loss of heterozygosity (0.63 [0.54–0.71], 0.76 [0.63–0.87]) for distinguishing mucinous from nonmucinous cysts, as well as the sensitivities and specificities of tests for *KRAS* mutations (0.59 [0.46–0.71], 0.78 [0.71–0.85]) and loss of heterozygosity (0.89 [0.78–0.96], 0.69 [0.60–0.76]) for differentiating malignant from benign cysts.* Conclusion*. Tests of *KRAS* mutations could confirm but not exclude a diagnosis of a mucinous or malignant pancreatic cyst.

## 1. Introduction

Pancreatic cysts comprise a pathologically heterogeneous class of lesions with many common clinical features [1, 2]. These cysts can be simple pseudocysts or cystic neoplasms, including benign lesions (e.g., serous cystadenoma), potentially malignant lesions (e.g., mucinous cystadenoma and intraductal papillary mucinous neoplasm (IPMN)), and malignant lesions (e.g., mucinous cystadenocarcinoma).

It can be difficult to differentiate pancreatic lesions on the basis of clinical presentation alone [3]. Radiologic imaging methods [4–8], such as CT, MRI, ultrasonography, and endoscopic ultrasonography (EUS), can aid in the characterization of such lesions as pseudocysts or as benign, potentially malignant, or malignant cysts [9, 10].

Cystic lesions of the pancreas have been investigated by EUS-guided aspiration of the cystic fluid and sampling of the cyst wall, septa, and mural nodules [11, 12]. A recent study concluded that cystic fluid analysis may aid in determining the optimal therapeutic strategy for certain patient groups (e.g., asymptomatic patients) [13]. Aspirated fluid can be analyzed by conventional tests, such as cytology, viscosity, extracellular mucin, tumor markers (e.g., carcinoembryonic antigen (CEA), cancer antigen (CA) 19-9, CA 15-3, and CA 72-4), and enzymes (e.g., amylase, lipase) [14–16]. However, despite its high specificity, cytology of the cystic fluid has low sensitivity for the differentiation of pancreatic cystic lesions. Moreover, although CEA and amylase tests of cystic fluid aspirates aid in the differentiation between mucinous and nonmucinous cysts [13], these tests are not diagnostic.

Molecular tests of the aspirated cystic fluid are particularly useful for detecting the accumulation of genetic mutations associated with lesion progression from early dysplasia to carcinoma. For example, molecular analyses of DNA amplified from biliary brush cells have been shown to provide highly sensitive and specific diagnoses [17]. Mutations in* KRAS* have been linked to cancer development. Other molecular tests analyze loss of heterozygosity (LOH), which refers to the loss of one parental copy of a gene (typically a tumor suppressor gene, for cancers).

Numerous studies have shown that molecular analyses of aspirates obtained by EUS-guided fine-needle aspiration (FNA) provide better detection and characterization of cystic lesions of the pancreas compared to other methods [18–26]. However, studies of EUS-guided FNA have generally included limited numbers of patients and have differed greatly in terms of test accuracy. Therefore, we performed a systematic review and meta-analysis of previous studies to determine the accuracy of molecular tests on pancreatic cystic fluid obtained by EUS-guided FNA. Specifically, we determined accuracy measures of tests for* KRAS* mutations, LOH, and DNA quantity to differentiate mucinous from nonmucinous and benign from malignant cysts of the pancreas.

## 2. Methods

### 2.1. Search Strategy

The Cochrane Library and electronic databases, including PubMed, EMBASE, Web of Science, BIOSIS, and LILACS, were searched for relevant articles published between 1990 (or 1994, for BIOSIS) and 2014. All searches were up to date as of May 2014. The following search terms were used: “pancreas,” “cyst fluid aspiration,” “cystic lesion or neoplasm of the pancreas,” “molecular analysis,” “sensitivity and specificity,” and “accuracy.” Experts in the field were contacted, and references from the retrieved primary and review articles were screened. Although no language restrictions were imposed initially, all articles chosen for final analysis were published in English. Letters to the editor and conference abstracts were excluded because they presented limited data.

### 2.2. Study Selection

A published study was included in the meta-analysis if (1) it utilized reference standards to make molecular diagnoses of pancreatic cystic lesions, (2) it analyzed at least 10 specimens, and (3) it provided sensitivity and specificity data or individual test values. Studies on the molecular analyses of pancreatic ductal adenocarcinoma or other neoplasms, such as pancreatic neuroendocrine tumor, were excluded. Two reviewers (X.R. G. and X.B. Z.) independently judged study eligibility while screening citations, and disagreements were resolved by consensus.

After independent review, 14 publications on the use of EUS-guided FNA for molecular analyses and diagnoses of cystic lesions of the pancreas were eligible for inclusion in the meta-analysis. Six papers were excluded because they were review articles on molecular analysis (3 articles) [27–29], they assessed pancreaticobiliary malignancy diagnoses from brush cytology samples (1 article) [17], they had insufficient information to calculate sensitivity or specificity (1 article) [26], and they had an alternative objective from our criteria (1 study) [30].

### 2.3. Data Extraction and Quality Assessment

Final articles were assessed and data were extracted independently by two reviewers (X.R. G. and X.B. Z.), who were blind to the publication details. Disagreements were resolved by discussion until consensus was reached. Data extracted from reports included participant characteristics, test methods, publication year, cut-off values, methodological quality, and outcome data, including sensitivity and specificity estimates or the numbers of true-positive, false-positive, false-negative, and true-negative results. Methods were assessed by using the Standards for Reporting of Diagnostic Accuracy (STARD) [31] and QUADAS-2, a revised tool for the quality assessment of diagnostic accuracy studies [32]. For each study, the following quality criteria were assessed: (1) cross-sectional or case-control design, (2) consecutive or random sampling of patients, and (3) prospective data collection. A study was arbitrarily defined as being of “high quality” if it met at least 17 of the 25 STARD criteria and had a low risk of bias on QUADAS-2, “low quality” if it met fewer than 13 of the 25 STARD criteria and had a high risk of bias on QUADAS-2, or “medium quality” otherwise.

### 2.4. Statistical Analysis and Data Synthesis

Standard methods were employed for the meta-analysis of diagnostic test evaluations [33–35]. Analyses were performed in the Meta-DiSc program for Windows (XI Cochrane Colloquium, Barcelona, Spain). The following parameters were calculated for each study: the sensitivity, defined as the true-positive rate; the specificity, defined as 1 − false-positive rate; the positive and negative likelihood ratios; and the diagnostic odds ratio. A random-effects model was used to calculate the pooled sensitivity, specificity, and other measures across studies, which are reported as the pooled values (95% confidence intervals, Cis).

A meta-analysis of the studies was performed by using the summary receiver operating characteristic (SROC) curve. Because the true- and false-positive rates are not independent, the SROC curve and area under the curve (AUC) represent the overall performance of the test. Unlike a traditional receiver operating characteristic plot that explores the effects of varying thresholds (i.e., cut-off points for determining positive results) of sensitivity and specificity in a single study, each data point in an SROC plot represents a separate study. Moreover, rather than showing the trade-off between sensitivity and specificity, regression lines in SROC curves show the true- versus false-positive rates for individual studies.

The *Q*-value, defined as the intersection point of the SROC curve with a diagonal line from the upper-left to the lower-right corner of the SROC space, corresponds to the highest common value of sensitivity and specificity for a test. This point does not indicate the best combination of sensitivity and specificity for a particular clinical setting but rather represents an overall measure of the discriminatory power of a test. Thus, the *Q*-value was used as a global measure of test efficacy.

Chi-square and Fisher's exact tests were used to detect statistically significant heterogeneity. A two-sided *P* value of less than 0.05 was considered statistically significant. To determine the factors responsible for heterogeneity in test accuracy, stratified (subgroup) analysis was used.

## 3. Results

### 3.1. Description of Included Studies

**Figure 1:**
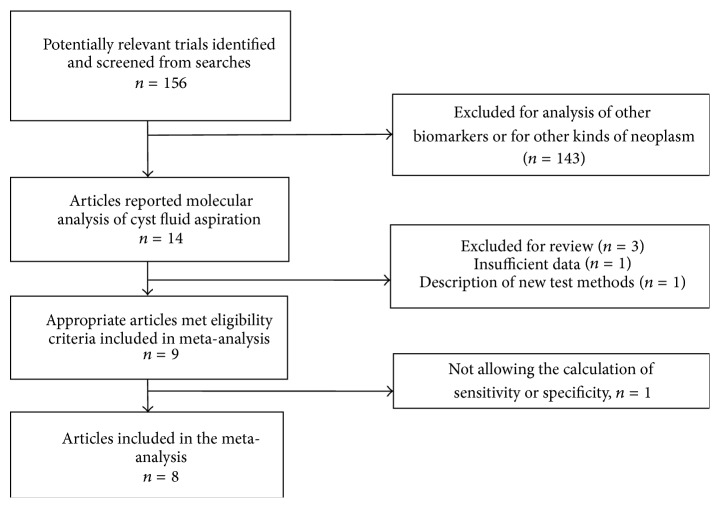
Study selection process.

Figure 1 outlines our study selection process. Eight articles including 428 patients were used for the meta-analysis, with an average sample size per study of 54 EUS-guided FNA specimens (range: 16–113 specimens). All studies used the same commercially available test for molecular analysis of the cystic fluid (PathFinderTG, RedPath Integrated Pathology, Pittsburgh, PA, USA).

### 3.2. Study Characteristics and Quality

The average interrater agreement between the two reviewers for items on the quality checklist was 0.85. Tables 1 and 2 present the descriptive data for each study, including study quality, sample size, and sensitivity and specificity estimates. In one study, patients were diagnosed by establishing a clinical consensus diagnosis. Cysts were classified as malignant, benign mucinous, or benign nonmucinous from histology or a combination of two of three concordant characteristics: EUS features, CEA level in cystic fluid, and cytology. Two studies diagnosed patients by follow-up histology, diagnostic cytology, or combined clinicopathologic interpretation, with most diagnoses being confirmed by the conventional gold standard (surgical pathology and malignant cytology). In the remaining four studies, surgical pathologic or malignant cytologic examinations served as the standard criterion.

As shown in Table 1, four of the eight studies (50%) were cross-sectional. Five studies (62.5%) collected samples from consecutive patients. Three studies (37.5%) were prospective. Overall, 37.5%, 25%, and 37.5% of the studies were of high, medium, and low quality, respectively (Table 2).

### 3.3. Diagnostic Accuracy of Tests of* KRAS* Mutations

**Figure 2:**
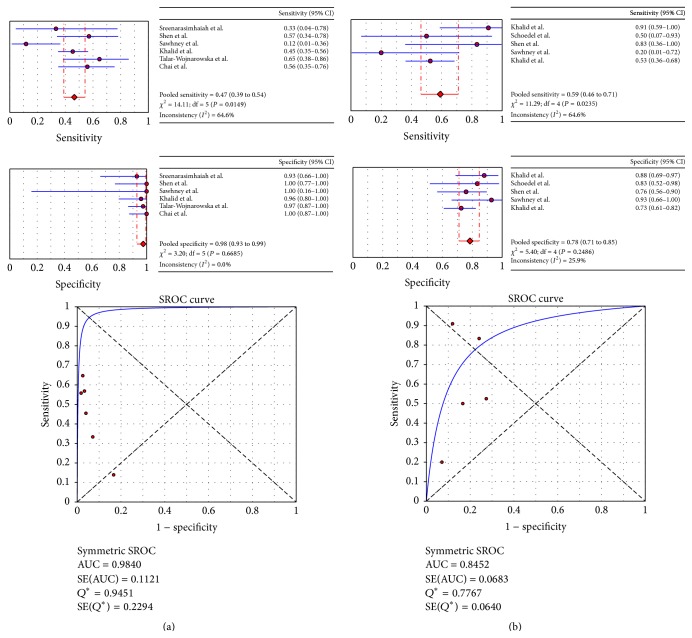
Forest plots of estimates of sensitivity and specificity and SROC in studies of K-ras mutations for the diagnosis of mucinous (a) and benign (b) cysts.

Six studies determined the accuracy measures of* KRAS* mutation tests in differentiating mucinous from nonmucinous and malignant from benign cysts (Figure 2). For the diagnosis of mucinous versus nonmucinous cysts, almost all studies had pooled specificity estimates close to 1.0 (0.98 (95% CI: 0.93–0.99)), whereas the pooled sensitivity estimates were lower and heterogeneous (0.47 (0.12–0.65); Figure 2(a)). The *Q*-value of 0.95, positioned near the desirable upper-left corner of the SROC curve (Figure 2(a)), and the AUC of 0.98 indicated a high level of accuracy. For the accuracy of tests of* KRAS* mutations in differentiating malignant from benign cysts, the pooled sensitivity averaged 0.59 (0.20–0.91) and specificity averaged 0.78 (0.73–0.93) (Figure 2(b)). The *Q*-value was 0.78 and the AUC was 0.85, indicating a moderate degree of accuracy (Figure 2(b)). Overall, this analysis demonstrated that tests of* KRAS* mutations have low sensitivity and high specificity in differentiating mucinous from nonmucinous and benign from malignant pancreatic cysts.

### 3.4. Diagnostic Accuracy of Tests of LOH

**Figure 3:**
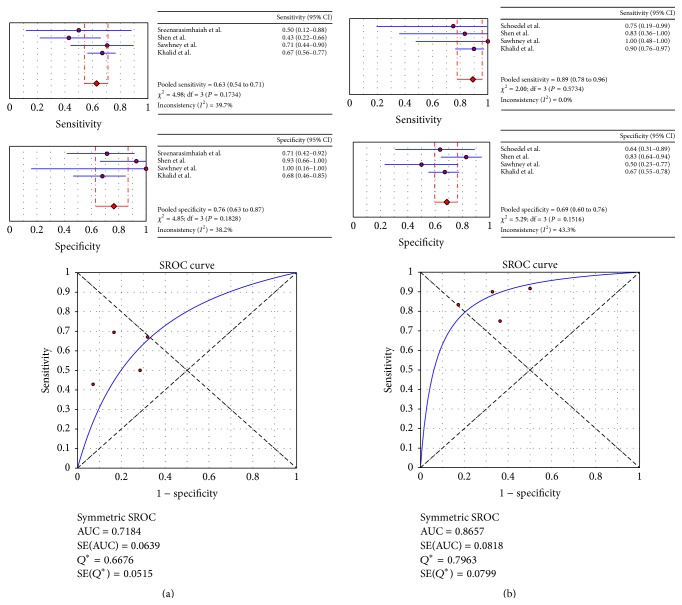
Forest plots of estimates of sensitivity and specificity and SROC in studies of LOH for the diagnosis of mucinous (a) and malignant (b) cysts.

Four studies determined the accuracy measures of LOH tests in differentiating mucinous from nonmucinous and malignant from benign cysts of the pancreas (Figure 3). For the differentiation of mucinous from nonmucinous cysts, low values were obtained for the pooled sensitivity (0.63 (0.43–0.71)) and specificity (0.76 (0.68–1.0)). The *Q*-value was 0.67, and the AUC was 0.72, indicating a low level of accuracy for differentiating mucinous from nonmucinous cysts (Figure 3(a)). In contrast, LOH tests accurately differentiated malignant from benign cysts, with a pooled specificity of 0.69 (0.50–0.83), pooled sensitivity of 0.89 (0.75–1.0), *Q*-value of 0.80, and AUC of 0.87 (Figure 3(b)).

### 3.5. Diagnostic Accuracy of Tests of DNA Quantity

Only three studies used DNA quantity to differentiate pancreatic cysts, which is an insufficient number of studies for meta-analysis. Also, these data could not be summarized by SROC curves.

### 3.6. Heterogeneity Analyses

Tables 3 and 4 show that many accuracy measures were homogeneous across studies, whereas some measures were heterogeneous. However, stratified analysis was unable to identify the sources of heterogeneity (data not shown).

## 4. Discussion

The pancreatic cysts lesions have been increasingly detected due to the use of cross-sectional imaging [36–38]. Discordant results have been reported from the many studies using EUS-guided FNA of pancreatic cysts for molecular analyses. We performed a meta-analysis to summarize evidence on the accuracy of molecular tests for the differential diagnosis of cystic lesions of the pancreas.* KRAS* mutation tests demonstrated high specificity and positive likelihood ratio values, but low and variable sensitivity values, in the differentiation of mucinous from nonmucinous and malignant from benign pancreatic cysts. The low sensitivity could be a result of low cellular loads in cystic fluid samples. LOH tests seemed to be more sensitive than DNA quantity tests in the differentiation of malignant from benign cysts. These results are in accordance with the publication showing that K-ras has a low sensitivity and a high specificity in the differentiation of benign and malignant pancreatic cysts [27]. However, this comparison should be interpreted cautiously because it is based on only a few, highly variable studies. Finally, although a stratified analysis could be used to determine the factors responsible for the heterogeneity in test accuracy, we had an insufficient number of studies to perform this analysis.

A pooled analysis was published regarding cyst fluid analysis in the differential diagnosis of pancreatic cystic lesions. The objective of the study is to investigate the value of cyst fluid analysis, including cyst-fluid concentrations of amylase, CA 19-9, or CEA in the differential diagnosis of benign (SCA, PC) from premalignant or malignant (MCA, MCAC) lesions. The study suggests that CEA > 800 ng/mL predicts MCA or MCAC and both CEA < 5 ng/mL and CA 19-9 < 37 U/mL suggest the presence of a SCA or PC, whereas amylase < 250 U/L makes a PC unlikely [13]. Also, in another meta-analysis, the diagnostic accuracy of cytology and CEA during EUS-FNA in differentiating mucinous and nonmucinous cystic lesions was studied. The study suggests that fine-needle aspiration has moderate sensitivity but high specificity for mucinous lesions [39]. These studies did not include molecular analyses like* KRAS* mutation and LOH tests as in our research. Theoretically, a combination of molecular analyses and cyst-fluid concentrations of amylase, CA 19-9, or CEA should increase the accuracy.

One limitation of our systematic review and meta-analysis is the lack of data in our included studies on the gains derived from using molecular analyses instead of conventional methods and other rapid tests. Only a few of the included studies directly compared molecular analyses with rapid tests, such as cytology and CEA tests [21–23]. As we did not include the terms “cytology” and “tumor markers” in our literature searches, our review cannot be used to identify the most accurate tests.

Very recently, a large multicenter study has been published investigating whether a combination of molecular markers and clinical information could improve the classification of pancreatic cysts and management of patients [40]. This study used a panel of molecular markers and also combined clinical features for the accurate classification. As we mentioned before, our research only included KRAS mutation and LOH tests. Because molecular genetics is a promising approach, more and more molecular analyses will be used in the further investigations of pancreatic cyst fluid studies. In the future, the optimum systematic reviews study design will incorporate more molecular analyses examination of cyst fluids taken at routine EUS-FNA. This could be most helpful for improved diagnostic accuracies, thereby allowing us to have a better segregation for benign and malignant diseases and tailor management plans.

In summary, our analysis suggests a potentially useful role for molecular analyses in the differential diagnosis of cystic lesions of the pancreas. Tests of* KRAS* mutations can confirm diagnoses of mucinous and malignant pancreatic cysts but should not be used to exclude a diagnosis due to their low sensitivity values. LOH tests had a low level of accuracy for differentiating mucinous cysts but were able to differentiate malignant from benign cysts accurately. We could not determine the accuracy of DNA quantity methods because of an insufficient number of studies. In conclusion, molecular analyses cannot replace conventional tests but should be used in parallel with clinical findings and conventional tests to make differential diagnoses of cystic lesions of the pancreas.

## 5. Conclusion

In conclusion, our study suggests that tests of* KRAS* mutations could confirm but not exclude a diagnosis of a mucinous or malignant pancreatic cyst.

## Figures and Tables

**Table 1:** Summary of included studies.

Study	Number of patients	Number of malignancies	Reference standard(s)	Cross-sectional design?	Consecutive sampling?	Prospective design?
Khalid et al., 2005 [18]	36	11/36 (31%)	Pathology, cytology	No	Unknown	Yes
Schoedel et al., 2006 [19]	16	4/16 (25%)	Pathology	No	No	No
Sreenarasimhaiah et al., 2009 [20]	20	9/20 (45%)	Pathology	No	No	No
Shen et al., 2009 [21]	35	6/35 (17%)	CCD	Yes	Yes	No
Sawhney et al., 2009 [22]	100	5/19 (26%)	Pathology	No	Yes	No
Khalid et al., 2009 [23]	113	40/113 (35%)	Pathology	Yes	Yes	Yes
Talar-Wojnarowska et al., 2012 [24]	56	NR	Pathology, cytology, follow-up	Yes	Yes	Yes
Chai et al., 2013 [25]	52	NR	Pathology, cytology, follow-up	Yes	Yes	NR

CCD: clinical consensus diagnosis; NR: not reported.

**Table 2:** Study quality and sensitivity/specificity for diagnosis.

Study	Quality	Mucinous versus nonmucinous			Malignant versus benign		
		*KRAS* mutations	LOH	DNA quantity	*KRAS* mutations	LOH	DNA quantity
Khalid et al., 2005 [18]	Low	NR	NR	NR	91/86	NR	NR
Schoedel et al., 2006 [19]	Low	NR	NR	NR	50/83	75/64	NR
Sreenarasimhaiah et al., 2009 [20]	Low	33/93	50/71	NR	NR	NR	NR
Shen et al., 2009 [21]	Medium	57/100	43/93	33/100	83/76	83/83	83/93
Sawhney et al., 2009 [22]	High	11/100	70/100	29/100	20/93	100/50	40/79
Khalid et al., 2009 [23]	High	45/96	67/68	45/68	53/73	90/67	75/86
Talar-Wojnarowska et al., 2012 [24]	High	65/97	NR	NR	NR	NR	NR
Chai et al., 2013 [25]	Medium	56/100	NR	NR	NR	NR	NR

Data are reported as the sensitivity/specificity of the different molecular tests for diagnosis of mucinous versus nonmucinous or malignant versus benign lesions. NR: not reported.

**Table 3:** Accuracy measures of molecular diagnostic analyses for the differentiation of mucinous from nonmucinous pancreatic cysts.

Test	Accuracy measure	Pooled summary measure^#^ (95% CI)	*P* value of heterogeneity test^*∗*^
*KRAS *mutation tests	Sensitivity	0.47 (0.39–0.54)	0.01^‡^
	Specificity	0.98 (0.93–0.99)	0.67
	Positive likelihood ratio	10.03 (3.72–27.06)	0.36
	Negative likelihood ratio	0.56 (0.43–0.73)	0.06
	Diagnostic odds ratio	19.69 (5.91–65.58)	0.27

*LOH *tests	Sensitivity	0.63 (0.54–0.71)	0.17
	Specificity	0.76 (0.63–0.87)	0.18
	Positive likelihood ratio	2.23 (1.35–3.66)	0.67
	Negative likelihood ratio	0.54 (0.42–0.70)	0.61
	Diagnostic odds ratio	4.65 (2.14–10.09)	0.77

^#^Random effects model. ^*∗*^Chi-square or Fisher's exact test for heterogeneity. ^‡^ *P* < 0.05.

**Table 4:** Accuracy measures of molecular diagnostic analyses for the differentiation of malignant from benign pancreatic cysts.

Test	Accuracy measure	Pooled summary measure^#^ (95% CI)	*P* value of heterogeneity test^*∗*^
*KRAS* mutation tests	Sensitivity	0.59 (0.46–0.71)	0.02^‡^
	Specificity	0.78 (0.71–0.85)	0.25
	Positive likelihood ratio	3.03 (1.79–5.11)	0.20
	Negative likelihood ratio	0.57 (0.32–0.99)	0.03^‡^
	Diagnostic odds ratio	7.45 (2.15–25.81)	0.11

LOH tests	Sensitivity	0.89 (0.78–0.96)	0.57
	Specificity	0.69 (0.60–0.76)	0.15
	Positive likelihood ratio	2.57 (1.85–3.57)	0.28
	Negative likelihood ratio	0.19 (0.09–0.39)	0.81
	Diagnostic odds ratio	15.62 (6.28–38.87)	0.81

^#^Random effects model. ^*∗*^Chi-square or Fisher's exact test for heterogeneity. ^‡^ *P* < 0.05.
